# Treatment approach for diabetes mellitus type 2 in a 70-year-old male: A case report

**DOI:** 10.1097/MD.0000000000042890

**Published:** 2025-08-01

**Authors:** Ndalela Ndalela, Annie Namataa Lingela, Limkile Mpofu

**Affiliations:** a Department of Post Graduate Studies, Human Sciences, Keystone University of Africa, Lusaka, Zambia; b Eye Department, Senanga General Hospital, Senanga, Western Province, Zambia; c Nursing Department, Senanga General Hospital, Senanga, Western Province, Zambia.

**Keywords:** depression, diabetes mellitus, diabetic foot, diabetic ketoacidosis, mental health

## Abstract

**Rationale:**

The case of the 70-year-old male with type 2 diabetes mellitus highlights the complexities involved in managing chronic conditions, particularly in low-resource settings. The need for holistic diabetes management policies and standard treatment guidelines is underscored by the patient’s deteriorating health and eventual death despite receiving multidisciplinary care. This case emphasizes the importance of structured approaches to diabetes management to improve patient outcomes and reduce mortality rates.

**Patient concerns:**

The patient presented with significant concerns, including general body weakness, inability to eat, and widespread body pains. These symptoms not only reflect the physical challenges associated with diabetes but also point to potential complications such as diabetic foot and acute depression, which can adversely impact the quality of life and overall health of individuals with diabetes.

**Diagnoses:**

This was a known diabetic patient who was diagnosed with diabetic foot and acute depression during his hospital stay. These diagnoses are critical as they represent common yet serious complications of diabetes that require immediate and effective management to prevent further deterioration of the patient’s health generally.

**Interventions:**

A multidisciplinary approach was employed in managing the patient’s condition, involving medical officers, clinical officers, nutrition technologists, eye health care providers, nursing caregivers, and psychosocial counsellors. This collaborative effort aimed to address the various aspects of the patient’s health, including therapeutic, surgical, physical, nutritional, and psychological needs, which are essential in managing diabetes effectively.

**Outcomes:**

Despite the comprehensive care provided, the patient unfortunately passed away on March 8, 2024. This outcome raises significant concerns regarding the effectiveness of the interventions implemented and highlights the challenges faced in managing diabetes in a low-resource setting. It also points to the need for ongoing evaluation and improvement of diabetes care protocols and policies.

**Lessons:**

This case serves as a critical reminder of the necessity for well-defined policies and treatment guidelines for diabetes management at all levels of healthcare. It emphasizes the importance of early intervention, continuous monitoring, and the integration of mental health support in the management of chronic diseases like diabetes, particularly in resource-limited environments.

## 1. Introduction

According to WHO,^[1]^ diabetes is a chronic metabolic disease characterized by elevated blood glucose levels, which leads to severe damage to the heart, eyes, blood vessels, kidneys, and nerves. The most common type 2 diabetes mellitus (DM), usually in adults, occurs when the body resists or does not produce enough insulin. Globally, diabetes is one of the leading causes of mortality and disability^[2]^ and accounts for the most significant rise in male deaths, with an 80% increase since 2000.

Management for DM type 2 is aimed at controlling blood sugar levels and reducing the risk of complications. This is achieved through lifestyle changes, medication, and regular monitoring.

## 2. Case presentation

A 70-year-old known diabetic male patient was brought to Senanga General Hospital on February, 17, 2024, presenting with general body weakness, not being able to eat food, and general body pains. He was earlier admitted to the same hospital on February 12, 2024 and discharged on February 15, 2024, just barely 2 days prior to his readmission on February 17, 2024.

He was a known diabetic and hypertensive patient since 2015. Drug history revealed that he was taking the following medicines to manage his conditions: nifedipine, moduretic, enalapril, metformin, and daonil. Physical examination findings revealed that he was unconscious, not able to talk, with labored breathing and a fruity smell. The vital signs were body temperatures ranging from 35 to 36.7°C throughout his admission, blood pressure was 139/71 mm Hg, pulse of 79 beats per minute, and respiratory rate was 22 breaths per minute. According to the fluid balance chart, there was no urine output. However, the patient was receiving normal saline intravenously. On this first admission, the following laboratory investigations were ordered. See Table 1 for laboratory results.

**Table 1:** Laboratory results of the 70-year-old diabetic patient during the initial admission from February 12, 2024 to February 14, 2024.

Investigation ordered	Result	Date	Remarks
Random blood sugar	23.5 mmol/L	February 12, 2024	–
Malaria RDT	Nonreactive	February 12, 2024	–
MPS	No malaria parasites seen.	February 12, 2024	–
Creatinine	181.8 µmol/L	February 12, 2024	50–104
Alanine transaminase	11.2	February 12, 2024	0–45
Urinalysis: color	Yellow	February 14, 2024	–
Turbidity	Clear	–	–
Pus cells	Nil	–	–
RBCs	2–3	–	–
Epithelial	3–5	–	–
Creatinine	169.1 µmol/L	February 14, 2024	Ref range 59–104.0
Alanine transaminase	14.0 µ/L	February 14, 2024	0–45.5

These results are interpreted as follows: all the highlighted result indicates an abnormally elevated parameter.

Random blood sugar: the result obtained was 23.5 mmol/L, confirming that the patient had abnormally elevated blood sugars. The test is used to diagnose diabetes, monitor treatment, and guide treatment in those who have already confirmed cases of diabetes. The normal random blood sugar is in the range of 5 to 7 mmol/L.

MPS and Malaria RDT are laboratory tests used to confirm malaria. In this case, these tests were ordered to exclude malaria because the patient had episodes of fever, since malaria is endemic in Zambia. NR means nonreactive, indicating that there were no malaria parasites seen.

Creatinine is a renal/kidney function test. In this case, this test result was extremely high on the first assessment, at about 181.8 µmol/L, and a second count of 169.1 µmol/L, indicating that there was kidney failure. The normal range is 50–104 µmol/L.

Alanine Transaminase is a test for liver function, and in this case, it was normal, indicating that there was nothing wrong with the liver. The normal range is 0 to 45.5 µ/L.

MPS = malaria parasite slide, NR = negative test result, RBCs = red blood cells, RDT = rapid diagnostic test.

## 3. Management and outcomes

A diagnosis of diabetic ketoacidosis (DKA) was made, and the patient commenced on normal saline 3 L to run for 12 hours, insulin soluble 18 IU in normal saline, insulin 7 IU subcutaneous hourly, and ceftriaxone IV OD. The nutrition technologist reviewed the patient and gave dietary guidelines such as encouraging sugar-free foods and low-fat diets, and further advised consumption of high-fiber foods. The patient was catheterized.

On February 13, 2024 at 12:33 hours, the random blood sugar (RBS) was ordered, and the result was 27.9 mmol/L, 25.0 mmol/L on the same day, while on February 14, 2024 at 06:00 hours, the RBS dropped to 15.6 mmo/L, following administration of 24 IU of insulin in 60 mL of normal saline and at 1800 hours, the RBS dropped to 8.0 mmol/L.

On February 15, 2024 at 00:00 hours, RBS dropped to 5.9 mmol/L and 5.8 mmol/L at 06:00 hours.

At this point, the patient was discharged from the hospital.

However, when the patient was readmitted, his blood pressure recorded was 140/76 mmHg, pulse 115 beats/minute, temperature 36.3°C, and RBS of 5.3 mmol/L. Physical examination revealed that he was generally weak and had foul-smelling pus discharging with excessive slough from his second right toe.

The patient exhibited unusual behavior of declining treatment and staying mute during the hour for medications. In addition, he attempted to remove all the tubing meant for management of his condition, such as a urinal catheter, oxygen tubing, and cannulas. He told his relatives that he would no longer want to live as he was suffering so much with his diabetes condition, which he referred to as “sugar disease.” Thus, the patient’s diagnosis of depression was based on clinical acumen and clinical picture as detailed here.

Therefore, with recurrent thoughts of death, further mental state examination by clinicians revealed psychomotor agitation, persistent sadness, and low mood.

A diagnoses of diabetic foot and acute depression were made.

Throughout his admission, he had temperature ranges of 36.3°C initially, then 39.0°C on his last day in the hospital, while his blood pressure range was 102/60 mm Hg to 168/78 mm Hg. SPO_2_ range was 96% to 98%.

The following table summarizes the laboratory investigations that were done during his second admission:

The therapeutic management of the patient was as follows:

On the admission day (February 17, 2024), he was commenced on Metformin 500 mg once a day (od), Daonil 5 mg od, Cloxacillin 500 mg four times a day per oral, Vitamin B complex 1 tab od, and Cefuroxime IV 2 g twice a day, which commenced on 2.3.24. One liter of Ringer lactate to run in 6 hours. Thereafter, all the antidiabetic medications were stopped, but wound cleaning of the second right toe continued as planned.

In due course, the necrotic toe was disarticulated after the family consented.

Insulin was reintroduced on March 6, 2024 as indicated in Table 2 for laboratory results above.

**Table 2:** Laboratory results for a 70-yr-old diabetic patient during hospital stay from February 17, 2024 to March 08, 2024.

Investigation ordered	Result	Date	Remarks
RBS	5.3 mmol/L	February 17, 2024	Done on admission
RBS	15.5 mmol/L	**February 22, 2024**	
	3.1 mmol/L	**March 4, 2024** – 14:00 h	
	2.3 mmol/L	**March 5, 2024 –** 02:00 h	
	16.1 mmol/L	18:00 h	
	16.1 mmol/L	**March 6, 2024 –**00:00 h	
	16.2 mmol/L	02:00 h	
	13.2 mmol/L	06:00 h	
	14.1 mmol/L	14:00 h	
	8.3 mmol/L	18:30 h	
	9 mmol/L	**March 7, 24** – 04:00 h	
	5.0 mmol/L	14:00 h	
	5.3 mmol/L	22:00 h	
	7.4 mmol/L	**March 8, 2024** – 04:00 h	
Creatinine	161.8 µmol/L	February 17, 2024	Ref range 59–104
ALT	19.0 µ/L	February 17, 2024	Ref range 0–45
Full blood count	–	–	Ref range
White blood count	12.90 × 10^3^	**February 20, 2024**	–
Red blood cell count	3.66 × 10^12^/L	–	4.13–5.57
Hemoglobin	11 g/dL	–	14.3–18.3
HCT	34.5%	–	41–52
MCV	94.3 fL	–	79.1–98.9
MCH	31.1 pg	–	27–32
MCHC	31.9 g/dL	–	32–36
Redcell distribution width	43.9%	–	11.6–14
Platelets	144 × 10^9^	–	137–373
Monocytes	4.6% 0.59 × 10^9^	–	0.08–0.61
Neutrophils	85% 10.95 × 10^9^	–	0.96–6.4
Urinalysis	Result	March 6, 2024	Remarks
Colour	Yellow		–
Turbidity	Clear	–	–
Pus cells	14–17	–	–
RBCs	20–23	–	–
Epithelial cells	4–7	–	–
Full blood count			
White blood count	19.30 × 10^3^	**February 27, 2024**	Ref range
Red blood cell count	3.11 × 10^12^/L	–	4.13–5.57
Hemoglobin	10.4 g/dL	–	14.3–18.3
HCT	29.1%	–	41–52
MCV	93.6 fl	–	79.1–98.9
MCH	33.4 pg	–	27–32
MCHC	35.7 g/dL	–	32–36
Redcell distribution width	44.1%	–	11.6–14
Platelets	116 × 10^9^	–	137–373
Monocytes	4.5% 0.87 × 10^9^	–	0.08–0.61
Neutrophils	90% 17.27 × 10^9^	–	0.96–6.4

The table above shows the laboratory results obtained for the patient while monitoring the treatment.

RBS was monitored on the dates and specific times indicated on the table. This was being done to guide and ascertain the effectiveness of treatment and to determine the prognosis. Hence, all highlighted random blood sugar fields indicate that the parameters are abnormally raised despite treatment.

Creatinine is a renal/kidney function test. In this case, the test result was extremely high on first assessment, at about 181.8 µmol/L, indicating that there was kidney failure. The normal range is 50 to 104 µmol/L.

Full blood count, also referred to as complete blood count; the blue highlighted field shows abnormally elevated WBCs count of 12.90 × 10^3^. This elevated white cell count indicated septicemia, that is, the presence of bacterial infection in the patient. Normal readings range from 4 × 10^3^ to 7 × 10^3^.

The fields highlighted in yellow show the red cell depression, HB of 11 g/dL, RBC of 3.66 × 10^12^/L. HCT of 34.5. These test results for HB, RBC, and HCT indicated that the patient was anemic, meaning he had less blood than normal in his body.

Red cell distribution width indicates that there is iron deficiency when it is high, as shown.

Urinalysis; this is the test used for the bio-microscopy analysis of urine. Test results showed that pus cells (14–17 per field) were high in urine, indicating a urinary tract infection. This is the patient who was using a urinary catheter.

As you look at the next full blood count parameters, you see almost a similar picture but they are more elevated than the previous indicating that either the treatment was missed in terms of managing the bacterial infection, since there was no culture and sensitivity test that was done to isolate the organism and the antibiotic sensitive to it.

The dates on which the listed laboratory investigations were conducted are highlighted in bold.

ALT = alanine aminotransferase, HB = hemoglobin, HCT = hematocrit, MCH = mean capsular hemoglobin, MCHC = mean capsular hemoglobin concentration, RBC = red blood cells, RBS = random blood sugar, WBCs = white blood cells.

Insulin Lente 30 IU subcutaneous AM, soluble 1.5 IU subcutaneous AM, Lente 1.5 IU PM, soluble 1.0 IU PM.

On March 8, 2024 at 05:30 hours, the patient died.

## 4. Discussion

This case study involves a diabetic patient who initially presented with ketoacidosis. This is a serious complication of diabetes that develops when the body cannot produce enough insulin, thereby breaking down fat as fuel and resulting in a buildup of acids (ketones) in the bloodstream.

According to the study by Hamdy,^[3]^ the following approach for the management of DKA should be considered and closely monitored.

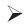
 Correction of fluid loss with intravenous fluids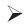 Correction of high blood sugars with insulin administration.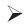 Correction of electrolyte imbalance, potassium loss in particular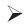 Correction of acid-base balance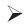 Treatment of concurrent infection in cases where infection is evident.

Mostly from a mix of hereditary, environmental, and lifestyle elements, type 2 DM has the main contributors, which are obesity and insulin resistance.

Obesity is a major risk factor as excess body fat – especially abdominal fat – can cause resistance here. Insulin resistance is aggravated by inflammation and elevated free fatty acids brought on by obesity.

Insulin resistance encourages uric acid retention that interferes with lipid metabolism, and second, hypertriglyceridemia can aggravate renal impairment, therefore lowering uric acid uptake and raising serum uric acid levels.^[4]^ At last, high ratios of uric acid to creatinine and triglyceride levels add to oxidative stress and low-grade inflammation, therefore establishing a feedback loop that aggravates both diseases. This case under review fits this biochemical transformational phase and the progress of inflammation that resulted in multiple complications, including diabetic foot. As seen from the laboratory results, the Creatinine levels spiked to around 181 µmol/L and RBS to around 23 mmol/L.

Chronic low-grade inflammation distinguishes type 2 diabetes (T2D), which is mostly caused by adipose tissue inflammation linked to obesity. This includes more macrophage infiltration and increased synthesis of pro-inflammatory cytokines like TNF-α and IL-6, hence disrupting insulin signaling and driving insulin resistance.^[5]^ Systemic markers – that is, C-reactive protein (CRP) – indicate increased T2D, hence show general inflammation. Furthermore, exacerbating cardiovascular issues and inflammatory mediators cause endothelial dysfunction,^[6]^ therefore emphasizing the critical role of inflammation in T2D pathogenesis.

According to the study by Aktas,^[7]^ in the clinical medicine journal, he emphasizes the importance of early detection of type 2 DM in its role in the prognosis of the disease. Early diagnosis is associated with a good prognosis and fewer complications of the disease. However, the prognostic nutritional index is attributed to the inflammatory sequelae of type 2 DM, and microvascular inflammations lead to serious inflammatory conditions that impact the physical, social, economic, and mental well-being of an individual.

If not properly controlled, DM type 2 is a chronic disease that can cause many consequences, including end-organ damage. Among the most important consequences is cardiovascular disease, since people with diabetes are more prone to heart attacks and strokes as a result of variables like high blood pressure.^[8]^

Another frequent consequence is neuropathy, which occurs when prolonged high blood sugar levels harm nerves and cause symptoms including pain, tingling, or numbness, most commonly in the hands and feet. If not sufficiently treated, this might cause major wounds, infections, or even amputations. Also, at risk for kidney disease^[9]^ called diabetic nephropathy, where the kidneys lose their filtering capacity and might advance to kidney failure needing dialysis or transplant – are diabetics. Furthermore, retinopathy can develop, which affects the retinal blood vessels and, if not treated with consistent eye exams and proper care, might cause blindness.

Because diabetes reduces blood flow and causes slow-healing wounds, which increases the risk of infections, patients may also suffer skin problems. Furthermore, just like in the case under review, the prevalence of depression and anxiety among diabetics aggravates the difficulty of adhering to treatment regimens and managing lifestyles.^[10]^

Hence, dealing with these issues calls for a multifarious strategy including frequent blood sugar level checks, a healthy lifestyle adoption, and continuous medical treatment to manage coexisting diseases. Early intervention and patient education are essential to avoid or reduce the long-term effects of type 2 diabetes.

Though the fluid therapy was instituted by infusing 3 liters of sodium chloride 0.9% fluid during his initial admission in this case study, the management approach still needed to be aggressive by monitoring the fluid output and the renal function tests to determine his renal efficiency. Renal efficiency can evaluate the need for further management, predominantly renal dialysis. In this case study, the Creatinine levels were constantly high during the patient’s stay in the hospital (highest 181.8 µmol/L and lowest 161.8 µmol/L).

Despite receiving intravenous fluids, the patient in this case study did not produce urine, which could have been a wake-up call to the attending health care team to investigate the renal failure. As per the fluid balance sheet, no urine output was recorded.

However, regarding the use of normal saline for fluid replacement therapy in DKA,^[11]^ some studies indicate that normal saline has been the traditional fluid of choice for both volume resuscitation and deficit replacement. Still, there are emerging reports of a higher risk of acute kidney injury and acidosis related to its chloride content. Jayashree et al^[11]^ still argue in their study that the mechanism of acute kidney injury is caused by renal vasoconstriction, which leads to reduced cortical tissue perfusion, renal interstitial edema, and intracapsular hypertension. In this case under review, sugar levels were controlled and dropped to 5.8 mmol/L within a space of 72 hours following the commencement of insulin subcutaneous 7 IU hourly, but this did not guarantee the patient’s safety and stability of blood sugar levels because the patient was discharged immediately after the blood sugars reached 5.8 mmol/L without necessarily monitor for constant blood sugar level stability.

After managing to control the blood sugar this far, the patient could have been kept in the hospital for the next 48 to 72 hours to allow monitoring by the managing team and health education on his general condition for such things as diet, how to live with it, how to administer medication, and so on. Chronically ill and DKA-related admissions pose a significant burden on Senanga General Hospital healthcare system, as it is a public health facility with resource limitations. This could probably be why some cases may not have prolonged hospital stays after restoring some of their health outcomes, just like in this case. Some literature reveals that some diabetic patients might suffer from vitamin B12^[12]^ and hence need supplements. In this case, under review, primary physicians considered supplements in the form of vitamin B complex; however, the patient did not benefit from it as his condition was in the terminal phase, and probably the infection arising from diabetic food could have disseminated throughout his blood, leading to a condition called septicemia.

According to the Zambian formulary,^[13]^ the drugs that are used for the management of diabetes are insulin and oral hypoglycemic medicines, including medications used in the treatment of hypoglycemia. There are 3 types of insulin: short-acting, intermediate, and long-acting. Short-acting insulin is the only type of insulin that can be administered intravenously during the treatment of diabetic emergencies, ketoacidosis, during surgery, or intravenous feeding. It can also be administered intramuscularly when the intravenous route is not available. When injected subcutaneously, it has a rapid onset of action (after 30–60 minutes), a peak action of 2 to 4 hours, and a duration of action of up to 8 hours. When injected intravenously, it has a short half-life of about 5 minutes, and its effect disappears within 30 minutes.

Intermediate and long-acting insulin, when given by intramuscular injection, has an onset of action of approximately 1 to 2 hours, a maximal effect at 4 to 12 hours, and a duration of 16 to 35 hours. The dosing regimen is variable but includes the following: A single morning injection of intermediate–action insulin. This may be combined with rapid-acting insulin to control mid-morning hyperglycemia from breakfast. The one–daily dose of intermediate-acting insulin is commonly used in elderly patients. A second dose of the intermediate-acting insulin before the evening meal may be given if adequate control is not achieved. This second dose can be given before bedtime to prevent nocturnal hyperglycemia if the peak effect occurs during sleep.^[13]^ Despite this vital information extracted from the Zambia National Formulary, management of diabetic ketoacidosis and in cases where there are persistently high uncontrolled blood sugars still pose a challenge in most of the health facilities in Zambia because the management approach is subjective, meaning it is dependent on the attending health care provider attending to the patient. There are no national treatment algorithms (protocols) for the management of diabetic emergencies, such as diabetic ketoacidosis, in the facilities. Above all, since diabetes is an electrolyte disorder, second-level hospitals must improve their laboratories to ensure all electrolyte profiles are conducted. In this case, being discussed, more than 3 physicians reviewed this patient, and each had their own approach towards its management, unlike in a situation where a treatment protocol was available and being followed.

Insulin can lead to hypoglycemia in diabetic patients, especially when their blood sugars are not monitored when they are out of the hospital. This is more likely in this case study because the patient was brought back to the hospital, presenting with general body weakness and unable to stand 2 days after his discharge. The RBS result was 5.3 mmol/L on admission. Though not justifying hypoglycemia, the caretakers must have attempted to give some food to their patient at home or on the way to the hospital, and this could have justified the RBS stated above; otherwise, it would have been much lower than that. The patient succumbed to diabetes complications of diabetic foot, and later, disarticulation was done. However, the complete blood count and differential counts done on February 27, 2024 showed a very high white blood count, depressed red cell line, neutrophilia, and monocytosis, highly suggestive of septicemia and some anemia. During the same period, the temperatures ranged from subnormal to 39°C. The intravenous antibiotic therapy only commenced on March 2, 2024. The literature recommends that treatment of concurrent infection in cases where the infection is evident must be initiated immediately. In this case, the management approach was more targeted at controlling blood sugar than the other co-morbidities. Depression emerged as one of the challenging factors in managing this case under review because counseling was not adequate and was probably being handled by general staff other than psychosocial counselors. According to literature^,[10]^ depression may also harm the immune system, making you more vulnerable to infections and diseases, leading to such things as irritability, anger, loss of interest in things that used to bring pleasure, headaches, preoccupation with death, trouble with memory and decisions, risk of heart attacks to mention but a few. In this case being discussed, the patient was withdrawn, lost interest in everything, including treatment, and declined to communicate with anyone, which was justification for intensive psychosocial counseling and support.

Psychosocial support is critical for people living with chronic diseases. It provides emotional, practical, and information to help people with chronic diseases such as DM cope with the challenges of living with their conditions. In this case being discussed, it was observed that the patient’s relatives were more concerned with the medical management of his condition rather than the psychosocial aspect. In addition, this case management focused more on the therapeutic approach. Lack of psychological support for this case is noted as a serious gap in care for the chronically ill.

According to the National Health Institute,^[14]^ being diagnosed with a chronic disease can be life-changing and worsen multiple physical and psychosocial consequences. Furthermore, a new diagnosis of chronic disease has been attributed to a crisis situation that leads to fear, anxiety, depression, and anger. However, in this case being discussed, the patient received inadequate 1 or 2 counseling sessions throughout his stay in the hospital without attention from mental health specialists.

In most of the low-resource settings, key human resources such as mental health care workers are inadequate, which poses a great challenge when faced with cases like the one under review.

The need for holistic, multidisciplinary treatment is underlined by the difficult interaction between physical problems and psychological anguish in diabetes, as shown in this case. The case is especially crucial in low-resource contexts like Senanga and other rural parts of Zambia, where mental health incorporation into chronic disease treatment is sometimes restricted.

## 5. Ethical considerations

Written consent was obtained from the relatives of the patient to have this case authored (the first son of the deceased represented the family). The purpose of the consent was well explained to the family, after which they consented to the use of information about the deceased for publication.

The case was presented to the hospital clinical and audit committee, where it was considered for publication, after which the Medical Superintendent (Hospital Executive Director) provided clearance for publication of the study.

The clearance letter and a signed consent form from the deceased’s family were uploaded and shared with the case reviewers.

## 6. Recommendations and key takeaway lessons

In managing diabetes with complications such as DKA, septicemia, and renal failure, a multidisciplinary approach is crucial^,[15]^ and at Senanga General Hospital, this includes close collaboration between the patient`s primary care physician, infectious disease specialists, and the laboratory for microculture sensitivity. In the case under review, no microculture and sensitivity tests were done due to the unavailability of a microculture and sensitivity laboratory owing to limited resources and infrastructure.

Primary clinicians (front line at OPD) were only engaged during a Major Doctors’ Round, which only takes place once a week.

Insulin therapy may be necessary for optimal glucose control in patients with renal failure. Still, in cases of septicemia, prompt and appropriate antibiotic therapy is essential, and the choice of antibiotics should consider the patient`s renal function to prevent further damage to the kidneys.^[16]^

## 7. Conclusion

This case highlights the importance of integrating mental health care services into chronic disease (such as DM type 2) management and establishing community-based education and support systems for long-term care.

Furthermore, having treatment protocols or guidelines in health facilities for managing diabetic emergencies and complications is critical.

However, multidisciplinary collaboration with the inclusion of mental health care service providers fosters quality interplay among the health care providers, resulting in good health outcomes.

Lastly, strict control of blood glucose levels, prompt treatment of infections, and proper management of renal function are essential for preventing further complications and improving the patient`s quality of life.

## Acknowledgments

The authors would like to thank Senanga General Hospital and all the relatives of the patient for allowing this case report to be published in the Medicine Journal.

## Author contributions

**Conceptualization:** Ndalela Ndalela, Annie Namataa Lingela, Limkile Mpofu.

**Data curation:** Ndalela Ndalela, Annie Namataa Lingela, Limkile Mpofu.

**Formal analysis:** Ndalela Ndalela, Annie Namataa Lingela, Limkile Mpofu.

**Funding acquisition:** Limkile Mpofu.

**Investigation:** Ndalela Ndalela, Annie Namataa Lingela, Limkile Mpofu.

**Methodology:** Ndalela Ndalela, Annie Namataa Lingela, Limkile Mpofu.

**Project administration:** Ndalela Ndalela, Limkile Mpofu.

**Resources:** Ndalela Ndalela, Annie Namataa Lingela, Limkile Mpofu.

**Software:** Ndalela Ndalela, Limkile Mpofu.

**Supervision:** Limkile Mpofu.

**Validation:** Ndalela Ndalela, Annie Namataa Lingela, Limkile Mpofu.

**Visualization:** Ndalela Ndalela, Annie Namataa Lingela, Limkile Mpofu.

**Writing – original draft:** Ndalela Ndalela, Annie Namataa Lingela, Limkile Mpofu.

**Writing – review & editing:** Ndalela Ndalela, Annie Namataa Lingela, Limkile Mpofu.
